# Vasopressin induced hyponatremia in infants <3 months of age in the neonatal intensive care unit

**DOI:** 10.3389/fped.2024.1465785

**Published:** 2024-10-02

**Authors:** Kavita Patel, Sharon Thomson, Meera Vijayan, Marjorie Makoni, Peter N. Johnson, Katy Stephens, Stephen B. Neely, Jamie L. Miller

**Affiliations:** ^1^ Department of Pharmacy, University Health, San Antonio, TX, United States; ^2^ Department of Pharmacy, Oklahoma Children’s Hospital at OU Health, Oklahoma City, OK, United States; ^3^ Department of Pharmacy, Lucile Packard Children’s Hospital Stanford, Palo Alto, CA, United States; ^4^ Department of Pediatrics, Section of Neonatology, University of Oklahoma College of Medicine, Oklahoma City, OK, United States; ^5^ Department of Pharmacy, Clinical and Administrative Sciences, University of Oklahoma College of Pharmacy, Oklahoma City, OK, United States; ^6^ Office of Instruction, Assessment, and Faculty/Staff Development, University of Oklahoma College of Pharmacy, Oklahoma City, OK, United States

**Keywords:** hyponatremia, infants, neonatal intensive care unit, neonates, vasopressin

## Abstract

**Objectives:**

Vasopressin is used for shock and acute pulmonary hypertension in the neonatal intensive care unit (NICU) and is associated with hyponatremia. The purpose of this study was to determine the incidence, severity, contributing risk factors associated with vasopressin-induced hyponatremia in neonates and infants <3 months of age in the NICU. The primary objective was to determine the incidence of hyponatremia (<130 mEq/L) and severe hyponatremia (<125 mEq/L). The secondary objectives were to compare clinical characteristics and the vasopressin regimen between those with and without hyponatremia.

**Methods:**

This retrospective cohort study included neonates and infants <3 months from 1/1/2017–12/31/2022 receiving vasopressin for >6 h. Analyses were performed using SAS v9.4, with *a priori* less than 0.05. A multiple variable logistic regression was employed to assess odds of hyponatremia.

**Results:**

Of the 105 patients included, 57 (54.3%) developed hyponatremia, and 17 (29.8%) were classified as severe hyponatremia. Overall, the median (interquartile range, IQR) gestational and postnatal age at vasopressin initiation were 35.4 (27–38.7) weeks and 2 (1–12) days. There was no difference in vasopressin dose, but duration of treatment was longer in those with hyponatremia. Higher baseline serum sodium was associated with decreased odds of hyponatremia [adjusted odds ratio (OR): 0.90 (95% CI: 0.83–0.99), *p* = 0.03], and increased vasopressin duration was associated with increased odds of hyponatremia [aOR: 1.02 (95% CI: 1.01–1.03), *p* < 0.001].

**Conclusions:**

Hyponatremia occurred in half of patients included. The pre-vasopressin sodium value and the vasopressin duration were independently associated with hyponatremia.

## Introduction

Vasopressin is used to stimulate V1 receptors leading to an increase in mean arterial blood pressure and systemic vascular resistance. Due to V1 receptor effects, it is commonly used in cases of vasodilatory shock or pulmonary hypertension with right ventricular dysfunction in the neonatal intensive care unit (NICU) (1–3). Vasopressin also has a role in regulating fluid balance through its stimulation of the V2 receptor (1). These receptors are located on renal tubular cells and cause decreased water excretion, leading to hyponatremia. Hyponatremia is associated with development of seizures and cerebral edema, but an additional concern that has been demonstrated in infants with hyponatremia is poor growth, increased mortality, and negative neurodevelopmental outcomes (4). Although hyponatremia is a recognized adverse effect of vasopressin therapy, there is a lack of published literature depicting the prevalence, specifically in the neonatal and infant population.

A number of case reports/series and studies have evaluated safety and/or efficacy of vasopressin in the pediatric intensive care unit (PICU), cardiac intensive care unit (CICU), and NICU have evaluated the impact on serum sodium (1, 5–22). Thirteen studies reported the incidence of hyponatremia, which ranged from 0% to 66% (1, 5–13, 15, 19–20). Most considered serum sodium concentrations below 125 mEq/L to be severe hyponatremia with ranges from <120–130 mEq/L (1, 6). Sodium concentrations were corrected with either 3% hypertonic saline or fluid restriction. However, there are no current studies that define risk factors for developing hyponatremia with vasopressin therapy in patients in the NICU. The purpose of this study was to characterize the incidence, severity, and contributing risk factors that are associated with vasopressin-induced hyponatremia in neonates and infants <3 months of age in the NICU.

## Methods

### Study design

This study was an Institutional Review Board approved retrospective, cohort study of neonates and infants <3 months of age who received vasopressin for ≥6 h while admitted to the NICU at a tertiary care academic children's hospital from January 1, 2017 to December 31, 2022. Patients were identified using the electronic database VigiLanz (VigiLanz Corporation, Minneapolis, MN). Patients were excluded if the indication for vasopressin administration was diabetes insipidus or cerebral salt wasting, if vasopressin was initiated following cardiac surgery, or if there were incomplete records of sodium concentrations. Only the first course of vasopressin was analyzed as it is unknown if repeated courses increase the severity or incidence of hyponatremia. If vasopressin was discontinued for more than 24 h, this was considered the completion of the first vasopressin course. Patients were classified into two groups based on the serum sodium concentrations during vasopressin therapy: hyponatremia (serum sodium ≤130 mEq/L) and no hyponatremia group.

### Study objectives and data collection

At the time of the study, there was no specified protocol for vasopressors, and the decision for vasopressor selection and dosing were at the discretion of the providers. Additionally, providers selected the initial intravenous fluids that patients received at the time of vasopressor initiation. At the study institution, standard practice for most patients admitted on the day of life 1 was to initiate a short-term neonatal amino acid solution containing dextrose 10%, amino acids 4%, and 20 mEq/L of calcium gluconate. In addition, providers will also initiate fluids for administration via the umbilical artery catheter and umbilical vein catheter that contain 0.225%–0.9% sodium chloride and dextrose 10%, respectively, at a rate of 0.5–1 ml/h to maintain line patency. Based on provider discretion, these patients are transitioned to parenteral nutrition containing 0–2 mEq/kg of sodium beginning on day of life 2.

Demographic data collected included: gestational age (weeks), postnatal age (days), biological sex, weight, and length. Vasopressin data collected included initial dose, all dosage changes, peak and final dose, and duration (hours). All serum sodium concentrations during the course of vasopressin administration were collected in addition to baseline serum sodium concentrations prior to the initiation of vasopressin therapy. To assess confounding variables, the number of patients receiving hydrocortisone were collected, as mineralocorticoids are known to impact the serum sodium and fluid balance (23, 24). In addition to this, administration of loop diuretics and chlorothiazide were collected as these agents have also been noted to cause hyponatremia and affect fluid balance (6, 25, 26). For patients that may have received intravenous bumetanide or furosemide, their dosing was converted to furosemide equivalent dosing in mg/kg (8, 25, 26). Additionally, the total daily sodium intake was calculated and included sodium from enteral feeds, enteral sodium supplements, intravenous fluids, parenteral nutrition, and intravenous medications. Total fluid intake and output were also recorded. The sodium content (mEq/kg) and total fluid intake and output (ml/kg) were calculated in 24-h increments for the day before sodium nadir, day of nadir, and day after nadir.

The primary objective of this study was to determine the overall incidence of hyponatremia from the onset of vasopressin. For this study, hyponatremia and severe hyponatremia were defined as <130 mEq/L and <125 mEq/L, respectively. The secondary objectives were to compare baseline and change in serum sodium concentrations, vasopressin peak dose and duration, and clinical characteristics between those with and without hyponatremia. Another secondary objective was to compare clinical characteristics and the vasopressin regimen in those with a serum sodium of 125–129 mEq/L vs. those with severe hyponatremia. An additional secondary objective was to identify patient-specific factors associated with development of hyponatremia.

### Statistical analysis

Descriptive statistics were employed. Inferential statistics included chi-square, Fisher's exact, or exact chi-square tests for nominal data and Wilcoxon 2-sample tests for continuous data, given the nonparametric distribution of the data. A multiple variable logistic regression model was used to determine the odds of hyponatremia with independent variables including vasopressin duration, baseline sodium concentration before vasopressin initiation, postnatal age, and gestational age. A generalized estimating equation (GEE) was added to the model with patients nested by year of treatment to account for any changes in vasopressin therapy and fluid management that may have occurred over time, and the exchangeable working correlation ρ was reported. Model diagnostics were performed by examining residuals and Cook's *d*. Model predicted sensitivity and specificity were reported. Analyses were conducted via SAS STAT version 9.4 (Statistical Analysis System, Cary, North Carolina). Tests of significance were two-tailed with significance defined as *p* < 0.05. Confidence intervals were reported with all model estimates [i.e., 95% Confidence intervals (CI)].

## Results

### Baseline demographics

During the study period, 216 patients receiving vasopressin were identified. A total of 111 patients were excluded for the following reasons: incomplete records (*n* = 58), and vasopressin administered for <6 h (*n* = 53). One hundred and five infants were included for analysis. Fifty-seven (54.3%) met the definition of hyponatremia (serum sodium <130 mEq/L); 17 (29.8%) of these met the study definition for severe hyponatremia (serum sodium <125 mEq/L). The remaining 48 patients were included in the no hyponatremia group.

Table 1 provides a comparison of the demographics of those with and without hyponatremia. There was no significant difference in the gender and weight between groups. The overall median [interquartile range (IQR)] gestational age was 35.4 (27–38.7) weeks, and the overall median (IQR) postnatal age was 2 (1–12) days. There was no significant difference between groups for gestational age, postmenstrual age, or postnatal age. Considering this study was conducted over a 6-year time-period, an attempt was made to compare the year that vasopressin was used to account for increased use of vasopressin and subtle changes in practice during the study timeframe at the study institution. There was no significant difference between the years that vasopressin was received between groups (*p* = 0.053).

**Table 1:** Comparison of clinical characteristics and demographics in those with and without hyponatremia.

Variables	Hyponatremia (*n* = 57)	No hyponatremia (*n* = 48)	*P*-value
	Number (%) or median (IQR)		
Demographics			
Males	31 (54.4)	27 (56.3)	0.848
Postnatal age (days)	2 (1–14)	1 (1–10)	0.069
Gestational age (weeks)	35.4 (26.7–38.6)	35.7 (28.9–38.9)	0.634
Postmenstrual age (weeks)	36.4 (29.4–39.1)	36.9 (29.8–39.2)	0.951
Weight (kg)	2.48 (1.25–3.46)	2.56 (1.25–3.24)	0.787
Year vasopressin initiated:			0.053
2017	6 (10.5)	8 (16.7)	
2018	14 (24.6)	3 (6.3)	
2019	8 (14.0)	10 (20.8)	
2020	9 (15.8)	7 (14.6)	
2021	15 (26.3)	9 (18.8)	
2022	5 (8.8)	11 (22.9)	
Sodium concentration data			
Baseline sodium (mEq/L)	136 (133–139)	137 (135–140)	0.060
Nadir sodium during vasopressin (mEq/L)	126 (124–127)	132 (131–136)	<0.001
Post-vasopressin nadir sodium (mEq/L)	133 (129–141)	136 (131–144)^a^	0.039
Percent change in sodium (%)	−7.3 (−10.7 to −5.3)	−2.8 (−4.7 to 0.8)	<0.001
Time to serum sodium <130 mEq/L (hours)	26.0 (12.0–33.0)	–	–

^a^ Post-vasopressin sodium values were only available in 46 patients.

### Changes in serum sodium concentrations and vasopressin regimen between groups

Table 1 also provides the data pertaining to serum sodium concentration obtained prior to and during vasopressin use. There was a significant difference in the number of patients who met the study definition of hyponatremia before vasopressin was initiated in the hyponatremia vs. no hyponatremia group, 23 (40.4%) vs. 9 (18.8%), *p* = 0.017. Overall, the median baseline serum sodium was 137 mEq/L, and there was no difference between the hyponatremia or no hyponatremia group. There was a significant difference in the median nadir serum sodium during vasopressin between those with and without hyponatremia, 126 vs. 132 mEq/L, *p* < 0.001. In addition to this, there was a significant difference in the median percent change of serum sodium from baseline to nadir between the hyponatremia and no hyponatremia group, −7.3% vs. 2.8%, *p* < 0.001. In the hyponatremia group, the time to their first serum sodium <130 mEq/L was a median of 26 h after initiation of vasopressin.

There was no significant difference in the median (IQR) initial vasopressin dose in milliunits/kg/min between those with and without hyponatremia, 0.2 (0.2–0.4) vs. 0.3 (0.2–0.5), *p* = 0.432. There was a numerically higher median (IQR) peak dose in the hyponatremia vs. no hyponatremia group, 0.9 (0.5–1.4) vs. 0.7 (0.4–1.0) milliunits/kg/min, but this difference was not significantly different (*p* = 0.173). The vasopressin dose was tapered down from the peak dose prior to discontinuation for a majority of patients. There was no significant difference in the median final dose between those with and without hyponatremia, 0.2 vs. 0.2 milliunits/kg/min, *p* = 0.311. Overall, the median duration of vasopressin was 37 h, and there was a significant difference in the median (IQR) duration between the hyponatremia and no hyponatremia groups, 40 (25–61) vs. 22 (15–45) hours, *p* ≦ 0.001. Of note, nine (8.6%) patients expired during vasopressin therapy including seven (12.3%) in the hyponatremia group and two (4.2%) in the control group, but this was not significantly different between groups (*p* = 0.296).

Table 2 provides a comparison of those who had severe hyponatremia (<125 mEq/L) vs. those who were hyponatremic with a serum sodium of 125–129 mEq/L. There was no difference in the initial, peak, or final median vasopressin dose between groups. However, there was longer median (IQR) duration of vasopressin between those with a serum sodium of 125–129 mEq/L vs. severe hyponatremia, 40 vs. 56 h, but this difference was not significantly different *p* = 0.219. In addition, there was no significant difference in the time to nadir between groups (*p* = 0.190). Overall, 41 (71.9%) of the patients with hyponatremia received a one-time intermittent infusion of 3% sodium chloride infusion to increase their serum sodium, but there was no difference between those with a serum sodium of 125–129 mEq/L vs. those with severe hyponatremia who received the 3% sodium chloride infusions, 29 (72.5%) vs. 12 (70.6%), *p* = 1.000.

**Table 2:** Comparison of vasopressin regimen and sodium concentration values in those with hyponatremia versus severe hyponatremia**.**

Variables	Hyponatremia (serum sodium 125–129 mEq/L) (*n* = 40)	Severe hyponatremia (serum sodium <125 mEq/L) (*n* = 17)	*P*-value
	Number (%) or median (IQR)		
Vasopressin regimen			
Initial vasopressin dose (milliunits/kg/min)	0.2 (0.2–0.4)	0.3 (0.3–0.4)	0.198
Peak vasopressin dose (milliunits/kg/min)	0.8 (0.5–1.5)	1 (0.7–1.5)	0.318
Final vasopressin dose (milliunits/kg/min)	0.2 (0.2–0.7)	0.3 (0.1–1.0)	0.777
Duration (hours)	40 (25–50)	56 (36–68)	0.219
Sodium concentration data			
Baseline sodium (mEq/L)	136 (133–139)	137 (133–139)	0.827
Nadir sodium during vasopressin (mEq/L)	127 (126–128)	122 (121–124)	<0.001
Post-vasopressin nadir sodium (mEq/L)	136 (130–142)^a^	130 (126–134)	0.031
Percent change in sodium (%)	−6.9 (−8.6 to −4.5)	−11.0 (−12.7 to −6.8)	0.001
Time to serum sodium <130 mEq/L (hours)	24.5 (11.0–31.0)	27.0 (18.0–41.0)	0.190

^a^ Available in 38 patients.

### Comparison of concomitant medications, sodium content, and fluid intake between groups

Table 3 provides a comparison of concomitant corticosteroids and diuretics that patients received. Overall, 83 (79.0%) received hydrocortisone; there was no significant difference in the number of patients in either group receiving concomitant hydrocortisone or the mg/kg dose of hydrocortisone 24 h prior to the sodium nadir. Only two (1.9%) patients received intravenous chlorothiazide, including one in each group. Twenty (19.0%) patients received an IV intermittent loop diuretic including 19 receiving furosemide and one receiving bumetanide. There was no significant difference in the number of patients receiving an intravenous loop diuretic or the mg/kg furosemide equivalents 24 h prior to the sodium nadir between groups.

**Table 3:** Comparison of concomitant medications in those with and without hyponatremia.

Variables	Hyponatremia (*n* = 57)	No hyponatremia (*n* = 48)	*P*-value
	Number (%) or median (IQR)		
Corticosteroids:			
Received corticosteroids during vasopressin therapy	46 (80.7)	37 (77.1)	0.168
Hydrocortisone IV dosing equivalents (mg/kg) 24 h prior to nadir	4.65 (2.7–8.4)	3.5 (2.4–6.5)	0.171
Chlorothiazide:			
Received chlorothiazide during vasopressin therapy	1 (1.8)	1 (2.1)	–
Chlorothiazide IV dosing (mg/kg) 24 h prior to nadir	6.5	45.0	–
Loop diuretics:			
Received loop diuretics during vasopressin therapy	12 (21.1)	8 (16.7)	0.474
Furosemide IV equivalent dosing (mg/kg) 24 h prior to nadir	2.2 (0.96–5.1)	3.5 (2.0–5.1)	0.700

Table 4 provides a comparison of the sodium content, fluid intake and output, and urine output between groups. The majority (*n* = 92; 87.6%) received parenteral nutrition, but there was no significant difference in the number of patients in the hyponatremia or no hyponatremia group that received parenteral nutrition, 50 (87.7%) vs. 42 (87.5%), *p* = 0.973. There was no significant difference in the median sodium intake in mEq/kg/day 24 h prior to nadir between groups (*p* = 0.115). However, there was a significant difference in the median sodium intake in mEq/kg/day in the day of nadir and the 24-h period post-nadir between those with and without hyponatremia, 9.0 vs. 4.4, *p* < 0.001, and 7.7 vs. 5.0, *p* = 0.007. There were no significant differences noted in the fluid intake and output and urine output in the 24 h prior to nadir, day of nadir, or 24 h after nadir between groups.

**Table 4:** Comparison of sodium intake and fluid intake and output in those with and without hyponatremia.

Variables	Hyponatremia (*n* = 57)	No hyponatremia (*n* = 48)	*P*-value
	Number (%) or median (IQR)		
Received total parenteral nutrition during vasopressin therapy	50 (87.7)	42 (87.5)	0.973
Sodium intake: (mEq/kg)			
24 h prior to nadir	4.0 (1.9–7.8)	3.4 (0.7–6.0)	0.115
Day of nadir	9.0 (5.1–13.1)	4.4 (2.9–7.0)	<0.001
24 h after nadir	7.7 (4.1–11.6)^a^	5.0 (2.8–7.5)	0.007
Fluid intake: (ml/kg/day)			
24 h prior to nadir	137.0 (118.0–167.0)	125 (105.0–158.0)^b^	0.106
Day of nadir	141.0 (110.0–164.0)	131 (107.0–156.0)	0.398
24 h after nadir	126.0 (100.0–152.0)^a^	124 (101.0–152.0)^c^	0.843
Fluid output: (ml/kg)			
24 h prior to nadir	64.4 (29.4–88.1)^d^	50.2 (29.6–80.2)^c^	0.288
Day of nadir	85.5 (47.8–135.1)	84.8 (50.5–118.2)^b^	0.568
24 h after nadir	96.2 (58.0–144.0)^e^	105.8 (57.6–154.0)^c^	0.496
Urine output: (ml/kg/h)			
24 h prior to nadir	2.8 (1.7–3.5)^d^	2.0 (1.4–3.4)^b^	0.077
Day of nadir	3.5 (2.1–6.2)	3.8 (2.1–5.1)^b^	0.568
24 h after nadir	4.0 (2.5–6.2)^d^	4.5 (2.6–7.1)^c^	0.500

^a^ Available in 55 patients.

^b^ Available in 47 patients.

^c^ Available in 46 patients.

^d^ Available in 54 patients.

^e^ Available in 53 patients.

### Multiple variable logistic regression analysis

A multiple variable logistic regression was performed to identify the odds of hyponatremia with several independent variables including vasopressin duration, baseline sodium concentration, postnatal age, and gestational age. The logistic model revealed that for each 1 h increase in the vasopressin duration, the adjusted odds of hyponatremia increased by 2% [adjusted Odds Ratio (aOR) 1.02; 95% CI: 1.01–1.03; *p* < 0.001]. Additionally, for every 1 mEq/L increase in the baseline serum sodium concentration, the adjusted odds of hyponatremia decreased by 10% (aOR 0.90; 95% CI: 0.83–0.99; *p* = 0.029). The postnatal age (aOR 1.01; 95% CI: 0.99–1.04; *p* = 0.428) and gestational age (aOR 0.97; 95% CI: 0.90–1.03; *p* = 0.314) were not associated with an increased or decreased adjusted odds of hyponatremia. Using the predicted values from the model and a cut point of 0.5, the model has a sensitivity for hyponatremia of 0.74 and specificity of 0.63. The GEE exchangeable correlation for year of treatment was weak positive (ρ=0.024).

## Discussion

To our knowledge, this is the largest study that has evaluated the incidence and risk factors of hyponatremia in neonates and infants <3 months of age receiving vasopressin in the NICU. Thirteen studies have evaluated the incidence of vasopressin-induced hyponatremia in critically ill pediatric patients (1, 5–13, 15, 19–20). Six of these included patients in the NICU setting (5, 12, 13, 15, 19, 20). The incidence of hyponatremia in patients in the CICU and PICU is 0%–66% vs. 30%–64% in the NICU (1, 4–13, 15, 19–20). Our study found that 54.3% of neonates and infants <3 months met the study definition of hyponatremia. As hyponatremia has been associated with poor weight gain and seizures in premature neonates, this clinically significant adverse event is important to recognize and identify opportunities for prevention and treatment (27).

Previous studies evaluating hyponatremia with vasopressin in the NICU, PICU, and CICU have had different definitions of hyponatremia including <125 mEq/L (*n* = 1), <130 mEq/L (*n* = 1), <134 mEq/L (*n* = 1), and <135 mEq/L (*n* = 7) (1, 5, 6, 9, 11, 12, 15, 19, 20). Eight studies did not have an explicit definition of hyponatremia (7, 10, 13, 16–18, 21). Our study definition of hyponatremia was ≤130 mEq/L. Only three previous studies had a separate definition of severe hyponatremia with either <125 mEq/L or <130 mEq/L (1, 11, 15). Only one of these evaluated premature neonates in the NICU setting, and they found 33% developed severe hyponatremia <125 mEq/L (15). Our definition of severe hyponatremia was <125 mEq/L, and we noted approximately 30% with severe hyponatremia. So, our findings seem consistent with their findings.

Several factors may have contributed to the development of hyponatremia including the type of maintenance fluids received at the time of vasopressin initiation, baseline serum sodium prior to vasopressin, dose and duration of vasopressin, and other concomitant fluids and medications. The majority of studies evaluating vasopressin-associated hyponatremia did not provide the type of intravenous maintenance fluids that their patients were receiving. Two studies noted that the majority of their patients received hypotonic maintenance fluids with either dextrose 5% with 0.225% sodium chloride or 0.45% sodium chloride, and as a result, 48%–49.4% of patients developed hyponatremia (1, 11). Another study by Bradford et al. (8) included patients <6 months in the CICU and noted that all patients received dextrose 5% with Ringer's lactate, and their incidence of hyponatremia was 15%. The majority of patients in our study were initiated on vasopressin within the first 2 days of life with an overall median postnatal age of 2 days. These patients were receiving the institution's short-term amino acid solution which does not contain sodium and possibly a sodium containing fluid running through the umbilical artery catheter which provides approximately 0.3–1.5 mEq/kg/day of sodium. One factor that could have contributed to a higher incidence of hyponatremia in our study compared to the previous studies is the lower provision of sodium in maintenance fluids. Another factor to consider is the baseline serum sodium prior to vasopressin initiation. It is not uncommon for neonates to have low or low normal serum sodium concentrations in the first 2 days of life due to the presence of increased extracellular water and minimal supplementation of serum sodium (28). An association with baseline serum sodium prior to vasopressin initiation and development of hyponatremia was noted in the conditional logistic regression analysis, with each 1 mEq/L increase in the baseline serum sodium associated with a decreased adjusted odds of hyponatremia of 10% when controlling for other variables.

Previous reports have investigated the impact of vasopressin dose and duration with hyponatremia. For shock, vasopressin dosing ranges from 0.17 to 8 milliunits/kg/min for critically ill infants and children (29). In our study, patients were initiated at 0.2–0.3 milliunits/kg/min of vasopressin, and we noted no difference between the median peak vasopressin dose between those with and without hyponatremia, 0.8 vs. 1 milliunits/kg/min. These findings are consistent to other studies, as two other studies that also evaluated vasopressin dosing found no significant difference in the peak dose in those with and without hyponatremia (1, 8). Extended duration of vasopressin has also been evaluated for an association of hyponatremia with vasopressin. Two previous studies evaluating vasopressin in infants and children in the CICU following cardiac surgery found that those with hyponatremia had a longer duration of vasopressin exposure than those without hyponatremia (1, 8). We also found a significant difference in the median duration of vasopressin between those with and without hyponatremia, 40 vs. 22 h, *p* = 0.001. Given that a number of other variables can be associated with hyponatremia, a conditional logistic regression was employed controlling for the baseline sodium before vasopressin initiation, postnatal age, and gestational age. We noted an association of duration of vasopressin and hyponatremia with the adjusted odds of hyponatremia increasing by 2% for each 1 h increase in vasopressin duration.

Previous studies have also assessed the impact of corticosteroids, diuretics, and concomitant fluids received along with vasopressin to assess the impact on hyponatremia. A previous study by Bradford and colleagues (8) evaluated infants <6 months of age receiving vasopressin in the CICU following cardiac surgery. They found a significantly higher number of patients that developed hyponatremia received chlorothiazide or loop diuretics compared to those who did not develop hyponatremia. Overall, we noted no difference in the cumulative dosing 24 h prior to nadir and the number of patients receiving either chlorothiazide or loop diuretics in patients with and without hyponatremia. It is likely that patients in the NICU may have received less diuretics as they were more premature and younger and did not have a cardiac origin to their shock. Other studies have evaluated the impact of corticosteroids on hyponatremia. Bradford and colleagues (8) noted a higher number of patients in the hyponatremia group received hydrocortisone vs. the no hyponatremia group, 75% vs. 45%, *p* = 0.028. We noted that the majority of patients in both groups received hydrocortisone for shock management and found no difference in the number receiving hydrocortisone between groups. We did not include the presence of corticosteroids and diuretics in our conditional regression model, but the previous study by Bradford and colleagues (8) noted that these variables were not associated with hyponatremia.

Several limitations must be noted with our study. First, this is a single-center study. Given the variability in fluid practices and other shock management, it is possible that incidence of hyponatremia could vary between institutions. Second, it is a retrospective design. We attempted our best to account for all enteral and parenteral fluids that patients may have received to account for the fluid and sodium intake at vasopressin initiation. However, given the retrospective nature, it is plausible that we may have not accounted for all sources of fluids that these patients received. Third, this study was conducted over a 5-year period. As clinicians have become more comfortable with the use of vasopressin in the NICU, it is possible that this may have influenced the incidence of hyponatremia as they may have become more proactive at fluid and sodium management around vasopressin initiation. However, we compared the year of vasopressin initiation and found no difference in the time-periods between those with and without hyponatremia. Fourth, as noted previously, there are varied definitions of hyponatremia in the literature; however, our selection of <130 and <125 mEq/L for hyponatremia and severe hyponatremia is in line with many of the studies evaluating vasopressin in older children. Last, we did not collect additional baseline demographics such as the number of patients who were mechanically ventilated or details pertaining to other vasopressor agents. These factors may provide additional details about the baseline severity of illness that patients in our cohort may have had. However, 80% of patients in this study were receiving hydrocortisone for hypotension at the time of vasopressin initiation, which could be a surrogate marker for a high acuity population, and there was no difference in use of corticosteroids or mg/kg/of corticosteroids in the 24 h prior to sodium nadir between groups.

Hyponatremia occurred in 54% of neonates and infants in the NICU setting, with one-third developing severe hyponatremia. The pre-vasopressin serum sodium concentration and the vasopressin duration were independently associated with severe hyponatremia. Clinicians should consider initiation of sodium containing fluids at the time of vasopressin initiation along with frequent electrolyte monitoring to prevent the development of hyponatremia in the NICU setting.

## Data Availability

The datasets presented in this article are not readily available because the dataset includes de-identified data collected retrospectively from the medical record according to the local IRB regulations. This dataset would not be available for review. Requests to access the datasets should be directed to Jamie Miller (jamie-miller@ouhsc.edu).
